# Artificial Intelligence- and Machine Learning-Assisted Subphenotyping for Personalized Immunotherapy in Sepsis

**DOI:** 10.3390/jpm16010028

**Published:** 2026-01-05

**Authors:** Evdoxia Kyriazopoulou, Eleni Karakike, Pavlos Myrianthefs

**Affiliations:** 1 2nd Department of Critical Care Medicine, Medical School, National and Kapodistrian University of Athens, 12462 Athens, Greece; ekyriazopoulou@windowslive.com; 2 Department of Critical Care, School of Nursing, National and Kapodistrian University of Athens, 14564 Athens, Greece

**Keywords:** sepsis, phenotypes, machine learning, precision medicine, immunotherapy

## Abstract

**Background/Objectives**: Sepsis heterogeneity limits advances in immunotherapy. Increasing use of artificial intelligence (AI) and machine learning (ML) attempts to turn multi-dimensional data into meaningful clusters, indicating biological mechanisms. We provide an overview of the existing evidence on AI-derived sepsis subtyping, exploring treatment response to available immune modulating therapies. **Methods**: On 1 October 2025, we conducted a structured search on all relative publications on MEDLINE and undertook a narrative review. **Results**: Multiple subphenotyping algorithms were identified, using clinical, biological, and omics data, across different cohorts, mainly through secondary analyses of randomized trials. The main classification was between hyper- and hypoinflammatory subphenotypes. Statins, corticosteroids, activated protein C, or thrombomodulin displayed differential effects on the outcome of these subphenotypes. **Conclusions**: Further research is required to prospectively validate findings and to offer pragmatic solutions to patients who need them the most. Issues of validity, equity, ethics, and feasibility are discussed.

## 1. Introduction

Sepsis is a heterogenous disease, involving multiple layers of variability, related to the pathogen, site of infection, comorbidities, host-immune response, and disease trajectory. Clinical trials targeting sepsis pathogenesis over decades have failed to capture this heterogeneity, leading to the lack of approved sepsis-specific therapies and the inability to improve sepsis outcomes despite 30 years of research. Sepsis management needs a paradigm shift of the trial design from prognostic (e.g., severity scoring-based) to predictive (e.g., mechanism-based) enrichment and treatment identification. This implies the identification of patient subgroups sharing common pathogenetic pathways, using simple or more complex biomarkers to assess the immune status [1,2,3,4,5,6].

Artificial intelligence (AI) and the science of machine learning (ML) attempt to identify patterns and generate knowledge from large datasets (e.g., data from the electronic health record -EHR, specific laboratory values, or physio markers) to subsequently cluster patients into subtypes for screening, diagnostic, and prognostic purposes and, most importantly, to select tailored interventions [7]. Specifically, ML is further divided into supervised and unsupervised. Supervised learning is focused on the mathematical function linking input data and a label; thus, it is mainly applied in studies aiming toward sepsis diagnosis and prognostication. Unsupervised learning is focused on describing the underlying structure or hidden patterns in a high-dimensional dataset; thus, it is mainly applied in studies aiming to categorize sepsis patients, a quite heterogeneous larger population, into smaller groups with distinct characteristics, namely “subgroups”, “phenotypes”, “subphenotypes”, “clusters” responding uniformly to targeted treatment. The most popular algorithms of unsupervised ML are principal component analysis (PCA), K-means clustering, hierarchical cluster analysis (HCA), latent class analysis (LCA), and latent profile analysis (LPA) [8].

High-throughput data, coming from the integration of multiple omics technologies (genomic, transcriptomic, proteomic, metabolomic profiling, and microbiome analysis), offer an unprecedented opportunity, as biomarkers, to describe and characterize activated pathogenetic pathways during sepsis. They may provide meaningful clustering into groups, possibly with a common response to certain targeted interventions; this could revolutionize sepsis treatment, offered to the most suitable candidate what is called personalized or precision medicine. As this technology becomes more widely available, multiple clustering efforts are reported, the validity, concordance, and clinical utility of which remains to be proven [9].

This study aims to summarize the existing evidence on AI/ML-assisted subtyping efforts in sepsis and associated treatment, with a focus on immunotherapy.

## 2. Materials and Methods

**Figure 1:**
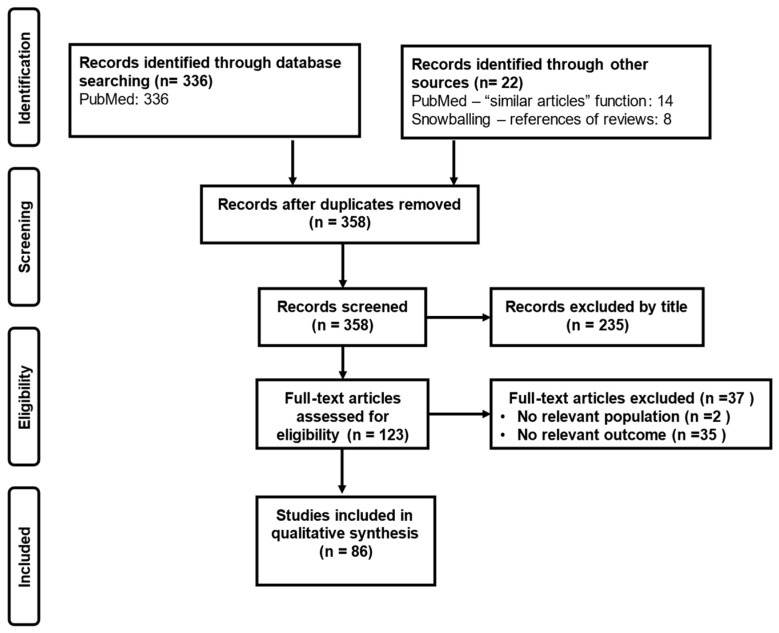
Study selection.

A search was conducted across MEDLINE [PubMed] on 1 October 2025, using the terms “artificial intelligence”, “machine learning”, “phenotyp*”, “subphenotyp*”, “subgroup”, “cluster”, “sepsis”, “critical illness”, “precision OR personalized”, “immunotherapy”, for studies published in English without publication time restriction. The full search string was as follows: (((artificial intelligence[Title/Abstract]) OR (machine learning[Title/Abstract])) AND ((((phenotyp*[Title/Abstract]) OR (subphenotyp*[Title/Abstract])) OR (subgroup[Title/Abstract])) OR (cluster*[Title/Abstract])) AND ((sepsis[Title/Abstract]) OR (critical illness[Title/Abstract]))) OR ((((((phenotyp*[Title/Abstract]) OR (subphenotyp*[Title/Abstract])) OR (subgroup[Title/Abstract])) OR (cluster*[Title/Abstract])) OR ((precision[Title/Abstract]) OR (personalized[Title/Abstract]))) AND ((immunotherapy[Title/Abstract]) OR (immune modulation[Title/Abstract])) AND ((sepsis[Title/Abstract]) OR (critical illness[Title/Abstract]))). We included observational studies (both prospective and retrospective) and randomized clinical trials, reporting on the subphenotyping/clustering of critically ill patients through machine learning/artificial intelligence algorithms. The use of the “similar articles” function was permitted, and references of the retrieved articles were screened to identify publications not captured by the initial search. Studies were selected if published in English, without publication time restriction. Articles not providing full text, not written in English, or not peer-reviewed, were excluded. The search was performed by two independent authors, and the same authors assessed all articles generated by the electronic search, by title, abstract, and complete text, to find those meeting the eligibility criteria. The study selection is shown in Figure 1.

To ensure language consistency throughout the manuscript, we adopted the consensus terms phenotype, subgroup, subphenotype, endotype, and treatable trait, as previously defined by experts in the field; Thus, a phenotype was defined as an observable set of clinical features; syndromes such as acute respiratory distress syndrome (ARDS) and sepsis could be considered phenotypes. A subgroup means any subset of patients within a phenotype, which may be defined using any cutoff in any clinical variable or measure (e.g., PaO_2_/FiO_2_-based severity classification of ARDS). A subphenotype is a distinct subgroup of a phenotype based on a shared set or pattern of observable or measurable properties, which can be reliably discriminated from other subphenotypes. An endotype is defined as a subphenotype, with distinct functional or pathobiological mechanisms, which may or may not be associated with a specific treatment response [8]. In this manuscript, distinct patterns in gene expression or metabolomic profiling are described as endotypes. A treatable trait is defined as a set of clinical characteristics and/or biomarkers, indicative of an underlying pathogenetic mechanism that responds to a specific intervention and may be present across multiple clinical syndromic diagnoses (e.g., hyperinflammation in ARDS and sepsis) [10]. Recently, it has been supported that critical illness should not be investigated through a syndromic approach but should instead be classified into distinct pathophysiological entities [1]. Syndromes, such as sepsis, ARDS, pancreatitis, or trauma may share common biological pathways ending up in similar subphenotypes, endotypes, and treatable traits.

## 3. Results

### 3.1. Artificial Intelligence/Machine Learning in Sepsis Subphenotyping

Here, we discuss all published literature regarding subgrouping or subphenotyping of critically ill patients with sepsis, based on algorithms of unsupervised ML, as summarized in Table 1. Due to the close relationship and overlap with sepsis, data on subphenotyping in other critical illness syndromes, such as COVID-19, hospital-acquired pneumonia (HAP), and ARDS are also presented, as they may inform and translate to a sepsis-specific context [1,10,11].

#### 3.1.1. Studies Using Clinical and Routine Laboratory Data in Machine Learning Models

In a pivotal study, Seymour et al. performed a retrospective analysis of the SENECA cohort, using data from 63,275 patients, who met the Sepsis-3 criteria within 6 h of hospital presentation, and derived four subphenotypes, named “clinical sepsis phenotypes” (α, β, γ, and δ); the α phenotype included patients with minimal organ dysfunction; the β phenotype referred to older patients with a higher comorbidity burden; the γ phenotype was characterized by higher inflammatory markers and pulmonary dysfunction; and the δ phenotype included patients with liver dysfunction and septic shock. Mortality was consistently higher in the δ phenotype, while differences in cytokine production among phenotypes suggested activation of proinflammatory pathways, coagulopathy, and endothelial dysfunction compared to the three other phenotypes. These subphenotypes were reproducible when retrospectively assessed in other international cohorts and randomized controlled trials (RCTs) [12]. They were also retrospectively identified, using latent class analysis, among critically ill COVID-19 patients [13]. Similarly, an independent study by Karakike et al. used a simplified (six-variable) algorithm for phenotype assignment, validating the presence, characteristics, and outcomes of the four clinical sepsis phenotypes, across a cohort of bacterial sepsis and severe COVID-19 [14]. In pediatric sepsis, Qin et al. applied consensus k-means clustering analysis to the PHENOtyping sepsis-induced Multiple organ failure Study (PHENOMS) dataset of 404 children and derived four computable phenotypes (PedSep-A, B, C, and D), very similar to the adult clinical sepsis phenotypes (α, β, γ, and δ); PedSep-D may benefit the most from early enrollment in personalized anti-inflammatory trials targeting thrombocytopenia-associated multiple organ failure and macrophage activation syndrome [15]. In another independent multi-center retrospective study, four acute respiratory failure (ARF) subphenotypes were identified among ICU patients who required mechanical ventilation due to sepsis-related ARF (A, B, C, and D); the ARF subphenotypes were directly comparable to the clinical sepsis phenotypes, with C being the less pathogenic and D displaying features of the δ phenotype. Data were similar when the pre- and post-COVID-19 periods were compared [16]. Although the reproducibility and generalizability of the clinical sepsis phenotypes in different settings is striking, the lack of an identifiable pathobiological mechanism underlying each subphenotype pointing to a specific biological process limits their use as treatable traits. The highly lethal δ phenotype, harboring both traits of coagulopathy and hyperinflammation, may be the most prominent to further investigate and target in prospective immunotherapy trials.

Various other distinct subgroups have been identified in ML studies, using clinical data from the large publicly available Medical Information Mart in Intensive Care (MIMIC)-II, -III, and -IV databases. Although there is less overlap with the traditional clinical sepsis phenotypes described above, these efforts also define distinct subgroups of patients with higher mortality, based on traits of higher comorbidity burden (such as in the β phenotype) or traits of coagulopathy (such as in the δ phenotype), as suggested above [17,18,19].

In some studies, longitudinal clinical data were used to develop subgroups, based on data trajectories [20,21,22,23,24]. Bhavani et al. used a previously validated temperature trajectory algorithm to classify 3576 patients into four temperature trajectory subphenotypes: hyperthermic slow resolvers; hyperthermic fast resolvers; normothermics; and hypothermics, with the highest mortality [25]. When combined with data on immune host biomarkers, these subphenotypes had distinct immune profiles, with hypothermics showing low cytokine expression, possibly compatible with immunoparalysis [26]. The same subphenotypes were validated among COVID-19 patients; hypothermics were associated with a hypercoagulable state and hyperthermic slow resolvers with a hyperinflammatory profile [27]. Another retrospective analysis of 3034 patients from the MIMIC-III database revealed seven subgroups, based on the systolic blood pressure (SBP) trajectory, with class 2 (low SBP, with no increasing trend) bearing the highest mortality [28]. More recently, among COVID-19 patients, both SBP and temperature trajectories were embedded in a classifier model to compute four subphenotypes; consistent with the previous findings, the hyperthermic–hypotensive (group A) and normothermic–hypotensive (group D) had the highest odds for 30-day mortality, with group A displaying higher inflammatory markers and bacterial co-infection and group D being characterized by coagulopathy and a higher prevalence of venous thrombo-embolic events [29].

Dynamic changes in subphenotypes, driven by interventions or by overtime changes in their composing parameters, are just as important as longitudinal data [30]. In a recent study, roughly 45% of patients changed subphenotype membership within the first 6 h of Emergency Department (ED) arrival [31]. Similarly, endotypes may also evolve over time and under the pressure of applied treatments; only 23.9% of patients remained within their initial endotype after 7 days [32,33,34]. Although stability to a specific favorable subphenotype or endotype may be a desirable treatment effect [32], subphenotype trajectories and transitions may also be useful in guiding interventions requiring daily titration, such as intravenous fluid or vasopressors administration. In a retrospective analysis of a cohort of patients with early septic shock from the ProCESS trial, five subphenotypes were identified, based on unique organ failure patterns. The highest-mortality group (H1) was characterized most notably by liver dysfunction and coagulopathy, while the second-high-mortality group (H2) exhibited primarily respiratory failure, neurologic, and renal dysfunction that persisted over time [35]. There was no difference in the outcome between high and low fluid administration in any of the groups identified.

Recently, ML has been used to identify patients at risk for cognitive impairment, recovering from sepsis. As sepsis is known to not end during hospitalization and to leaving chronic sequelae (post-sepsis syndrome), this study defined three clusters of different cognitive dysfunction, associated with differences in the administered opioid treatment, apart from female sex, older age, and comorbidities [36].

Following the paradigm of sepsis, ML algorithms have also been used for phenotyping other conditions of the critically ill, such as severe COVID-19 pneumonia, revealing consistently a hyperactive (more severe clinical alterations, higher inflammatory and coagulation markers, organ failure, and mortality) and a hypoactive (adaptive) subphenotype [37,38]. Similarly, in HAP, two clusters, namely HAP subphenotype 1 and 2, were derived; HAP subphenotype 2 was associated with higher disease severity, more prominent respiratory microbiome dysbiosis, higher levels of proinflammatory cytokines, and higher mortality [39].

#### 3.1.2. Studies Using Biomarkers in Machine Learning Models

Several studies use key biomarkers of the host immune response as classifiers, alone or in combination with clinical data. Madushani et al. assessed a wide variety of biomarkers in blood and urine, in addition to clinical data, obtained within 24 h in a cohort of surgical sepsis patients. Two clusters were identified: a more severe one, cluster I (early disrupted homeostasis), presenting a higher comorbidity burden, higher organ dysfunction, and high inflammatory biomarkers and associated with higher mortality and chronic critical illness and cluster II (early preserved homeostasis), which was associated with recovery [40]. In an interesting study from Uganda, unsupervised clustering methods were applied to 14 soluble host immune mediators, reflective of key domains of sepsis immunopathology, among patients with suspected sepsis. In parallel, whole-blood RNA-sequencing data were included from a consecutive subset of patients. Two immune subphenotypes (S1 and S2) were defined; S1 included more anti-inflammatory and endothelial-protective mediators (sTNFR1, IL-10, Ang-1), while S2 was coordinated around more proinflammatory and chemotactic (MIP-1α/CCL3, MIP-1β/CCL4, TNF-α) and endothelial-destabilizing (Ang-2) mediators. The proinflammatory S2 was associated with higher clinical severity and 30-day mortality. RNA-sequencing data reinforced this two- subtype partition, with endotype T2 (relevant to S2) exhibiting higher proinflammatory immune activation, T-cell exhaustion, and metabolic reprogramming and consistently worse outcomes, such as severe HIV-associated tuberculosis, extensive organ dysfunction, worse functional status, and higher mortality [41]. Bracht et al. analyzed a prospective multi-center sepsis cohort using plasma proteomics to describe and characterize sepsis plasma proteome subtypes. Four subtypes with different sepsis severity and mortality rate were identified. At the proteome level, the subtypes were characterized by distinct molecular features [42]. The same study group combined clinical data with proteomics longitudinally and ended up with three clusters. The identified clinical phenotypes reflected varying degrees of sepsis severity and were mirrored in the plasma proteome, showing the gradual consumption of complement and coagulation factors with increasing sepsis severity [43].

Perhaps the most widely validated example of biomarker-guided subphenotyping, comes from ARDS. Using clinical and biological data from 1022 patients included in two ARDS randomized controlled trials (ARMA trial and ALVEOLI trial), Calfee et al. applied latent class modeling to introduce two ARDS subphenotypes (also termed “molecular phenotypes”): the “hyperinflammatory”, characterized by higher plasma concentrations of inflammatory biomarkers (high levels of interleukin (IL)-6, IL-8, soluble tumor necrosis factor receptor one (sTNFR1)), a higher prevalence of vasopressor use, sepsis, and higher mortality, and the “hypoinflammatory”, showing higher levels of protein C and bicarbonate [44]. Ever since, these phenotypes have been reproduced and validated using biological data only, clinical data only, molecular data, and a combination of clinical and biological data through a standardized ML classifier model [45,46,47,48]; they have also been identified in sepsis with or without ARDS [49,50]. On a gene expression level, these subphenotypes differ: the hyperinflammatory type is characterized by the activation of pathways related to the innate immune response, motility, and energy metabolism, including oxidative phosphorylation, glycolysis, and cholesterol biosynthesis, but also PD-1, PD-L1 cancer immunotherapy, Rho signaling, and IL-8 signaling. The hypoinflammatory phenotype is enriched for pathways implicated in the T-cell response, PD-1 (programmed cell death protein 1), and IFN signaling and glucocorticoid and mineralocorticoid synthesis [48,50]. These phenotypes demonstrate distinct air space biology; hyperinflammatory ARDS is characterized by an increased interferon-stimulated gene expression and T cell activation in the lungs [51].

To summarize, subphenotyping using biomarkers is more informative when it is supported by molecular or clinical data indicating alterations in specific biological pathways, rather than by markers of severity. Every effort should be made to integrate multi-level data into recognizable pathobiological processes, to guide future treatment options.

#### 3.1.3. Studies Using Transcriptomics in Machine Learning Models

Combinations of ML algorithms and bioinformatics with the transcriptome as input have provided at least three distinct attempts of endotyping among sepsis patients. Davenport et al. performed genome-wide transcription profiling of samples of peripheral blood leucocytes coming from sepsis patients with community-acquired pneumonia and described two sepsis response signature (SRS) groups, namely SRS1 and SRS2. SRS1 was associated with higher mortality and features of immunosuppression, such as endotoxin tolerance, T-cell exhaustion, and downregulation of human leucocyte antigen (HLA) class II [52]. Scicluna et al., using bulk leucocyte RNA sequencing, defined four distinct endotypes, namely MARS 1–4, with MARS 1 having the worst outcome and a decreased expression of genes related to key innate and adaptive immune cell functions, such as Toll-like receptor, nuclear factor κB (NFκB1) signaling, antigen presentation, and T-cell receptor signaling, functions also altered in the SRS1 endotype. In contrast, MARS 3 was associated with a lower SOFA score and lower mortality and showed a significant association with the SRS2 [53]. Sweeney et al. identified three endotypes, “inflammopathic”, “adaptive”, and “coagulopathic”. The “adaptive” was the most common subtype associated with adaptive immune activation and low mortality; the “inflammopathic” was characterized by innate immune activation and high mortality; and the “coagulopathic” was characterized by clinical evidence of coagulopathy, as well as high mortality [54]. The presence of these endotypes has been also validated in COVID-19 pneumonia [33]; however, no “immunosuppression” cluster was described. Seeking generalizability, a consensus framework was developed by the three expert groups; using data from the MARS and GAINS cohorts, SRS 1–2, MARS 1–4, and the “inflammopatic”, “adaptive”, and “coagulopathic” endotypes were reclassified into “consensus transcriptomic subtypes (CTS) 1–3”, with CTS1 displaying features of classical inflammatory and endothelial activation, CTS2 heme disturbances, abnormal fibrinolysis, and higher mortality, and CTS3 with interferon signaling pathway activation, milder disease severity, and lower mortality [55]. Similar attempts of suphenotyping and endotyping have been made in pediatric populations. Wong et al. described three subclasses of genome-wide expression profiling, derived from whole blood-derived RNA (A, B, and C), corresponding to clinically relevant phenotypes [56].

Recently, transcriptomic modeling has become more multi-dimensional and comprehensive, including single-cell sequencing, immune cell population analysis, and epigenomic profiling. Zhang et al., interrogating variations in immune cells, molecules, and signal-transduction pathways, validated the presence of two endotypes, immunity A and B, corresponding to immunoparalysis and immunocompetent status, respectively, with the former (immunity A) associated with higher mortality [57]. Similarly, Zhou et al., using seven different publicly available transcriptome datasets from 1692 sepsis patients, including whole-genome, single-cell, and immune cell infiltrates, defined two distinct clusters, A and B. The Inflammatory cluster (A) was associated with high infiltration of macrophages and worse prognosis and functionally characterized by upregulation of the Toll-like receptor signaling pathway. In the Adaptive cluster (B), the T cell receptor signaling pathway was upregulated. Single-cell RNA sequencing analysis confirmed the functional profiling [58]. In another study, such data were used to specifically target the mechanism of cuproptosis (a novel cellular death process) and related genes. The authors described three sepsis subgroups, according to differential expression of these genes, with group B showing a lower overall expression of high-risk cuproptosis-related genes, less immune cell dysfunction, and better prognosis [59]. Heterogeneity in epigenomic changes, such as lactylation activity among patients with sepsis-associated acute respiratory distress syndrome (ARDS), enabled classification into low- and high-lactylation activity phenotypes, with distinct transcriptomic profile and differences in circulating immune cell populations [60].

As the complexity rises, numerous immune endotypes have been proposed for sepsis, the similarities and differences of which remain unclear. Recently, the SUBSPACE consortium evaluated the overlap of existing immune endotypes in sepsis across > 7074 samples from 37 independent cohorts and developed cell-type-specific gene expression signatures to quantify dysregulation within immune compartments. Myeloid and lymphoid dysregulation were associated with disease severity and mortality across all cohorts, also observed in patients with ARDS, trauma, and burns, suggesting a conserved mechanism across various critical illness syndromes [61]. In other words, the previously used terms “immunosuppression” and “hyperinflammation” may represent different states of immune dysregulation on a cellular level, the degree of which is the driver of mortality, rather than the state itself.

### 3.2. Precision Treatment for Artificial Intelligence/Machine Learning-Derived Subphenotypes in Critical Illness

#### 3.2.1. In Acute Respiratory Distress Syndrome (ARDS)

Calfee et al. was the first to present data on differential treatment responses by phenotype in critically ill patients in 2014. In a secondary analysis of the ALVEOLI cohort, the authors traced a difference in mortality rates by phenotype when different ventilation strategies (high positive end-expiratory pressure [PEEP] vs. low PEEP) were applied [44]. Similarly, a retrospective analysis of the eICU as derivation and MIMIC-IV as validation databases identified three different clusters of sepsis-associated ARDS, with different responses to high PEEP [62]. On the contrary, Serpa Neto et al. performed a secondary analysis of the PROVHILO trial, comparing high vs. low intra-operative PEEP in the operating room; there was no interaction between phenotype and PEEP level for the development of postoperative pulmonary complications [63].

#### 3.2.2. In Sepsis

Differences in mortality rates by ARDS phenotype (hyperinflammatory and hypoinflammatory) were documented in the FACTT RCT comparing liberal vs. conservative fluid administration strategies but not in the VASST RCT comparing low-dose vasopressin vs. norepinephrine in patients with septic shock [45,49]. Liu et al. described four subphenotypes of critical illness through ML algorithms in a large population (*n* = 8145) coming from datasets from the US, Europe, and China. Phenotype A comprised the subgroup of younger patients with the mildest disease severity; phenotype B the most common group of older patients with significant acid–base abnormalities and low white blood cell count; phenotype C presented hypernatremia, hyperchloremia, and hypercatabolic status; and in phenotype D, patients suffered from the most severe multiple organ failure. The beneficial fluid balance threshold intervals of the subphenotypes were different [64].

Shen et al. first reported the effect of the sepsis phenotype on the treatment of vasoactive drugs. Specifically, four phenotypes A–D were observed; phenotype A was highly sensitive to the dosage and time of norepinephrine equivalence (NEE, captured hourly for up to 72 h to record both the time of use and dosage); phenotype B showed a high relative risk of hospital mortality and low-dosage safety; phenotype C was primarily dosage-dependent; and phenotype D was characterized by severe multi-organ failure and hemodynamic instability, and the NEE dosage–mortality risk curve was U-shaped and dangerous [65]. Another review, written by leading experts in the field, has summarized the differential treatment effect by subtype in sepsis and ARDS [10].

Early administration of antimicrobials is considered key in sepsis management with ascending mortality with each hour of delay [66]. The only randomized trial on this subject, the Prehospital Antibiotics Against Sepsis (PHANTASi) trial, did not find significant mortality benefits from early antibiotics. In a recent secondary analysis, the investigators performed K-means clustering and ended up with three clusters based on age, heart rate, and temperature. There was a significant interaction between age and the benefits of early antibiotics even after adjustment for several other confounders [67].

### 3.3. Artificial Intelligence/Machine Learning-Derived Sub Phenotypes and Sepsis Immunotherapy

Immunotherapy may become the third pillar of sepsis treatment, along with early appropriate antimicrobial treatment (with source control where indicated) and organ support [68]. Thus, the differential effect of immunotherapy by sepsis subtype is of the utmost importance in sepsis management.

The evidence supports a pleiotropic effect of statins, including immunomodulating properties, mediated by the inhibition of isoprenoids, which act as intracellular signaling molecules. The HARP-2 trial, comparing simvastatin to placebo in ARDS patients, concluded that, among the previously described molecular ARDS phenotypes (hypo- and hyperinflammatory), the hyperinflammatory subphenotype had improved survival with simvastatin compared with a placebo [69]. On the contrary, the SAILS trial randomized sepsis associated-ARDS patients to rosuvastatin or a placebo, and no treatment effect was observed with rosuvastatin by phenotype [70].

A focused update of guidelines on the use of corticosteroids published in 2024 provided a conditional recommendation to administer corticosteroids for patients with septic shock [71]. However, the data remain inconclusive as to whether the clinical benefit is universal or limited to specific patient populations [72]. In a post hoc analysis of the VANISH RCT, randomizing patients with septic shock to hydrocortisone or placebo, patients were assigned to the already described SRS1 and SRS2 endotypes. An interaction between assignment to hydrocortisone or a placebo and the SRS endotype was found; hydrocortisone use was associated with increased mortality in patients with an SRS2 phenotype [50]. In a similar strategy, upon re-analysis of the VANISH RCT, corticosteroid use was associated with differential treatment effects by molecular ARDS phenotype assignment: the mortality was similar for patients with the hyperinflammatory phenotype, who were treated with hydrocortisone or a placebo (both 41%), but it was higher for patients with the hypoinflammatory phenotype receiving hydrocortisone than for those receiving placebo (44% vs. 10%), suggesting harm from corticosteroids in immunosuppressed subphenotypes. Moreover, a significant overlap was observed between the hypoinflammatory molecular phenotype and SRS2 endotype [50]. This is currently being prospectively investigated in the RECORDS RCT, a multicenter, placebo-controlled, biomarker-guided, and adaptive Bayesian design basket trial, assigning to a biomarker stratum 1800 adults with community-acquired pneumonia, vasopressor-dependent sepsis, septic shock, or acute respiratory distress syndrome. Among strata, the SRS2 endotype is used. In each stratum, patients are randomly assigned to receive a 7-day course of hydrocortisone and fludrocortisone or a placebo, and the primary outcome is 90-day death or persistent organ dysfunction [73]. Recently, a retrospective multicenter proof-of-concept study was published exploring the effects of corticosteroids in patients with sepsis, through a target trial emulation framework stratified by subphenotypes of predicted organ dysfunction trajectory (estimated through 72 h SOFA scores). The Rapidly Improving subgroup had an increased risk of mortality, a longer time to ICU discharge, and a longer duration of ventilation with steroid treatment, while the Rapidly Worsening group showed a decreased risk of mortality, a shorter time to ICU discharge, and a shorter duration of ventilation associated with steroid use [74].

Among other immune-modulating treatments, recombinant human thrombomodulin (rhTM) has anti-inflammatory and anticoagulation activities, and it has been suggested as an adjunct therapy for patients with sepsis, particularly those with sepsis-induced coagulopathy. In a secondary analysis of three multicenter registries on ICU patients with sepsis or septic shock in Japan, four subphenotypes were derived through ML, using the platelet count, PT-INR, fibrinogen, fibrinogen/fibrin degradation products, and D-dimer as predictors. Cluster dA had features of severe coagulopathy, severe organ dysfunction, and high mortality. Cluster dB had severe disease with moderate coagulopathy. Clusters dC and dD had moderate and mild disease with and without coagulopathy, respectively. Thrombomodulin was associated with a lower mortality only in cluster dA [75,76].

In a secondary analysis of the PROWESS-SHOCK trial, investigating recombinant human activated protein C (drotrecogin alfa activated-DrotAA) vs. a placebo in septic shock, a significant treatment interaction effect was observed with ARDS molecular phenotype assignment; DrotAA was associated with higher mortality in the hypoinflammatory and lower mortality in the hyperinflammatory subphenotype, compared with a placebo [49].

Ilofotase alfa, human recombinant alkaline phosphatase, has been shown to exert reno-protective properties and has been administered in patients with sepsis-associated acute kidney injury (AKI). In a secondary analysis of the REVIVAL RCT assigning patients with sepsis-associated AKI ilofotase alfa vs. a placebo, two subphenotypes were detected, based on the disease severity and organ dysfunction. Subphenotype 1 displayed relatively lower disease severity and less pronounced renal and pulmonary dysfunction, whereas subphenotype 2 exhibited higher severity and creatinine, with lower eGFR and bicarbonate levels. Ilofotase alfa significantly reduced major adverse kidney events up to day 90 for subphenotype 2, but not for subphenotype 1 patients [77].

The benefits from convalescent plasma therapy for COVID-19 has been inconsistent in clinical trials. In a secondary analysis, the REMAP-CAP investigators showed that patients with COVID-19 could be classified into three subphenotypes, based on multiple blood protein biomarkers (cytokines, chemokines, endothelial); one with elevated type II and II effector immune responses, another with exaggerated inflammation, and a third with variable biomarker patterns. Of those, only subphenotype 1 had better outcomes with convalescent plasma therapy compared with usual care [78].

A summary of studies assessing the effect of immunotherapy by subtype is provided in Table 2. In our view, studies where the heterogeneity of treatment effect is explained by the targeting of a specific mechanism (mediator effect) are to be preferred over strategies where the disease severity or organ dysfunction is used as a classifier.

**Table 1:** Summary of studies using machine learning for subphenotyping of the critically ill.

Study	Setting	Input Data	Method Used	Subphenotypes	Clinical Relevance
Seymour CW, 2019 [12]	Sepsis	Clinical and routine biological variables	K-means clustering, confirmation with latent class analysis	Phenotypes α, β, γ, δ (“sepsis clinical phenotypes”)	α phenotype: lower age, minimal organ dysfunction, and lower mortality β phenotype: older age, more chronic illness, and renal dysfunction γ phenotype: more inflammation and pulmonary dysfunction δ phenotype: more liver dysfunction, septic shock, and highest mortality
Aldewereld ZT, 2022 [35]	Septic shock	Clinical and routine biological variables	Hierarchical clustering	Clusters L1, L2, M, H1, H2	High-risk 1 (H1): higher age, multiple organ dysfunction, especially cardiac and respiratory failure; high mortality High-risk 2 (H2): younger age, liver dysfunction, and coagulopathy; higher levels of IL6, angiopoietin-2, thrombomodulin, and vWF; higher mortality Moderate risk: older age, pneumonia, and neurologic dysfunction Low-risk 1 (L1): younger age, fluid-refractory, few other organ dysfunctions, and low mortality Low-risk 2 (L2): younger age, fluid-responsive, and low mortality
Hu C, 2022 [17]	Sepsis	Clinical and routine biological variables	K-means clustering	Subphenotypes A and B	Subphenotype B: higher levels of lactate, glucose and creatinine, WBC, sodium, and heart rate and lower PaO_2_/FiO_2_ ratio; mortality higher
Guo F, 2022 [18]	Sepsis	Clinical and routine biological variables	K-means clustering	Clusters C_1, C_2, C_3, C_4	C_4: septic coagulation patients with the worst prognosis
Zador Z, 2019 [19]	Sepsis	Clinical and routine biological variables	Latent class analysis	Six subgroups: cardiopulmonary; cardiac; young; hepatic/addiction; complicated diabetics; uncomplicated diabetics	Mortality and organ dysfunction most prevalent in the hepatic/addiction subgroup
Miao S, 2025 [20]	Sepsis	Clinical and routine biological variables	Latent class mixed models	Eight leukocyte trajectory subtypes	Differences in mortality rates
Lei M, 2022 [22]	Critically ill—Trauma	Clinical and routine biological variables	Random forest, gradient boosting machine, decision tree, and support vector machine	Persistent critical illness (PerCI) and no-PerCI	PerCI: immunosuppressive state with worse nutritional status, more electrolyte imbalance and infection-related comorbidities, and more severe illness scores
Xu Z, 2022 [23]	Sepsis	SOFA score	Hierarchical agglomerative clustering	Rapidly worsening; delayed worsening; rapidly improving; and delayed improving phenotype trajectory	Rapidly worsening: higher comorbidity burden, acidosis, and visceral organ dysfunction; highest in-hospital mortality Rapidly improving: vasopressor use without acidosis
Xie Y, 2025 [24]	Sepsis	Clinical and routine biological variables	Group-based trajectory modeling	Four PDW trajectory groups: PDW rapidly increasing; low PDW stable; moderate PDW stable; high PDW	Variations in inflammatory marker levels and clinical outcomes
Bhavani SV, 2024 [26]	Sepsis	Clinical data: temperature; validation with host immune biomarkers	Group-based trajectory modeling	Hyperthermic slow resolvers, hyperthermic fast resolvers, normothermics, hypothermics	Hypothermic: higher mortality rate, lower levels of most pro- and anti-inflammatory cytokines
Bhavani SV, 2024 [29]	Critically ill—COVID-19	Clinical data: vital signs: oral temperature, heart rate, respiratory rate, and systolic and diastolic blood pressure	Group-based trajectory modeling	Groups A–D	Group A: high temperature, heart rate, respiratory rate, and hypotensive; higher mechanical ventilation and vasopressor use Group B: high temperature, heart rate, respiratory rate, and hypertensive Group C: low temperature, heart rate, respiratory rate, and normotensive Group D: low temperature, heart rate, respiratory rate, and hypotensive; higher mortality
Fernández-Gonzalo S, 2020 [36]	Post-sepsis syndrome	Clinical and routine biological variables	K-means clustering	Three clusters K1–K3	K1: severe cognitive impairment in speed of processing (92%) and executive function K2: moderate-to-severe deficits in learning-memory, memory retrieval, speed of processing (36.4%), and executive function K3: normal cognitive profile in 89% of patients Differences in opioid treatment in K1 vs. K2 and K3
Chen H, 2021 [37]	Critically ill—COVID-19	Clinical and routine biological variables	K-means clustering	Hyperactive and hypoactive phenotypes	Hyperactive: higher mortality and organ dysfunction
Velez T, 2023 [38]	Critically ill—COVID-19	Clinical and routine biological variables	Gradient-boosting machine algorithm	Phenotypes 1 and 2	Phenotype 2: older, elevated markers of inflammation and increased risk of ICU, sepsis, and mortality
Martin FP, 2025 [39]	Critically ill—HAP	Clinical and routine biological variables	Hierarchical clustering on principal components	PHOENYCS subphenotypes 1 and 2	Subphenotype 2: microbiome dysbiosis, higher levels of proinflammatory cytokines, and higher mortality
Choudhary T, 2024 [16]	Sepsis-associated ARF	Clinical and routine biological variables	K-means clustering	Phenotypes A, B, C, D	Differences in mortality rates Differences in treatment effects associated with high PEEP strategy
Bai Y, 2022 [62]	Sepsis-associated ARDS	Clinical and routine biological variables	K-means clustering	Cluster 0, Cluster 1, and Cluster 2	Cluster 0: lowest mortality and fewer laboratory abnormalities Cluster 1: highest mortality and most laboratory abnormalities Cluster 2: moderate mortality rate and longest ICU stay Different effect with higher PEEP
Madushani RWMA, 2022 [40]	Surgical sepsis	Clinical variables and biomarkers in blood and urine	Hierarchical clustering	Clusters I (early disrupted homeostasis) and II (early preserved homeostasis)	Cluster I: higher severity scores, chronic cardiovascular and renal disease, septic shock, and higher mortality and incidence of chronic critical illness
Cummings MJ, 2022 [41]	Suspected sepsis	Host immune mediators and transcriptomics	Hierarchical clustering	Subtypes 1 and 2 and respective endotypes T1 and T2	S2 (and T2): higher proinflammatory, chemotactic, and endothelial-destabilizing mediators, T-cell activation and tolerance, and neutrophil, monocyte/macrophage, NK, Th1, and dendritic-cell chemotaxis; more extensive organ dysfunction; severe HIV-related infections; higher mortality
Bracht T, 2025 [42]	Sepsis	Proteomics	K-means clustering	Clusters 0, 1, 2, 3	Cluster 0: 100% mortality Cluster 1: activation of the adaptive immune system Cluster 2: acute inflammation, low Ig levels Cluster 3: proteome baseline
Bracht T, 2025 [43]	Sepsis	Proteomics + clinical variables	Principal component analysis	Clusters A, B, C	Cluster C: higher mortality, acute liver failure and high-grade AKI, high inflammation, and proteome features of complement and coagulation factor consumption
Calfee CS, 2014 [44]	ARDS	Clinical variables and biomarkers	Latent class analysis	Hyperinflammatory (phenotype 2) vs. hypoinflammatory (phenotype 1) or ARDS molecular phenotypes	Hyperinflammatory (phenotype 2): higher plasma levels of IL-6, IL-8, sTNFr-1, and PAI-1; higher heart rate and total minute ventilation; lower systolic blood pressure, bicarbonate, and Protein C; higher prevalence of vasopressor use, sepsis prevalence, and higher mortality
Sinha P, 2023 [49]	Sepsis	Clinical variables and biomarkers	Latent class analysis	Hyperinflammatory (class 2) vs. hypoinflammatory (class 1) or sepsis molecular phenotypes	Hyperinflammatory: higher levels of IL-8, IL-6, sTNFr-1, creatinine, and bilirubin; lower platelets, serum bicarbonate, and protein C; higher mortality
Zhou W, 2022 [58]	Sepsis	Transcriptomics	K-means clustering	Adaptive subtype vs. inflammatory	Inflammatory subtype: high infiltration of macrophages, worse prognosis
Zhang S, 2020 [57]	Sepsis	Transcriptomics	Principal component analysis	Immunoparalysis vs. immunocompetent endotype	Immunoparalysis endotype: higher mortality
Wang Y, 2025 [60]	Sepsis-associated ARDS	Transcriptomics + clinical variables	K-means clustering	High- vs. low-lactylation activity phenotypes	Distinct transcriptional patterns, signaling pathways, drug sensitivity, and immune infiltration Six molecular biomarkers (ALDOB, CCT5, EP300, PFKP, PPIA, and SIRT1) differentiate between the two lactylation-based phenotypes
Davenport EE, 2016 [52]	Sepsis due to CAP	Transcriptomics	Hierarchical clustering followed by K-means clustering	SRS1 and SRS2	SRS1: immunosuppressed phenotype (endotoxin tolerance, T-cell exhaustion, and downregulation of HLA class II), higher 14-day mortality
Scicluna BP, 2017 [53]	Sepsis due to CAP	Transcriptomics	Unsupervised consensus clustering	MARS 1–4	MARS 1: impaired pattern recognition, cytokine and lymphocyte signaling, and antigen presentation; increased heme synthesis; higher mortality MARS 2: increased pattern recognition and innate signaling MARS 3: enhanced adaptive immune function (T-cell signaling) MARS 4: raised interferon signaling
Sweeney TE, 2018 [54]	Sepsis	Transcriptomics	Combined mapping of multiple clustering algorithms (communal)	Inflammopathic, adaptive, coagulopathic	Adaptive: adaptive immune activation and low mortality Inflammopathic: innate immune activation and high mortality Coagulopathic: coagulopathy and high mortality
Wong HR, 2009 [56]	Pediatric septic shock	Transcriptomics	Unsupervised hierarchical clustering, followed by K-means clustering	Subclasses A, B, C	Subclass A: younger patients with higher illness severity and higher mortality rate. Signaling pathways relevant to the adaptive immune system, glucocorticoid receptor signaling and zinc metabolism were repressed in subclass A

Abbreviations: AKI, acute kidney injury; ARDS, acute respiratory distress syndrome; ARF, acute respiratory failure; CAP, community-acquired pneumonia; HAP, hospital-acquired pneumonia; HLA, human leucocyte antigen; ICU, intensive care unit; PAI-1, plasminogen activator inhibitor-1; PDW, platelet distribution width; PEEP, positive end-expiratory pressure; SOFA, sequential organ failure assessment; SRS, sepsis response signature; sTNFr-1, soluble tumor necrosis factor receptor-1; vWF, von Willebrand factor; WBC, white blood cells.

**Table 2:** Summary of studies evaluating treatment effect by phenotype in the critically ill.

Study	Setting	Original Trial	Treatment Under Investigation	Subphenotypes	Treatment Response by Subphenotype
Calfee CS, 2018 [69]	ARDS	ALVEOLI	High PEEP vs. low PEEP	Hyperinflammatory and hypoinflammatory	Hyperinflammatory and high PEEP: lower mortality and more ventilator-free days than with low PEEP
Sinha P, 2020 [45]	ARDS	FACTT	Liberal vs. conservative fluid strategy	Hyperinflammatory and hypoinflammatory	Hyperinflammatory and conservative fluid lower mortality than with liberal fluid strategy
Sinha P, 2018 [70]	Sepsis-associated ARDS	SAILS	Rosuvastatin vs. placebo	Hyperinflammatory and hypoinflammatory	No difference
Calfee CS, 2018 [69]	ARDS	HARP-2	Simvastatin vs. placebo	Hyperinflammatory and hypoinflammatory	Hyperinflammatory and simvastatin: lower mortality
Sinha P, 2023 [49]	Septic shock	VASST	Low-dose vasopressin vs. norepinephrine	Hyperinflammatory and hypoinflammatory	No difference
Sinha P, 2023 [49]	Septic shock	PROWESS-SHOCK	Recombinant human activated protein C vs. placebo	Hyperinflammatory and hypoinflammatory	Hypoinflammatory and activated protein C: higher mortality
Serpa Neto A, 2018 [63]	Intra-operative ventilation	PROVHILO	High PEEP vs. low PEEP	Reactive and uninflamed	No difference
Liu P, 2023 [64]	Critical illness	Observational data	Fluid administration strategy	Phenotypes A–D	Different beneficial fluid balance threshold intervals
Shen J, 2025 [65]	Sepsis	Observational data	NEE	Phenotypes A–D (different from the study by Liu P et al.)	Phenotype A: highly sensitive to dosage and time of NEE; phenotype B: low-dosage safety; phenotype C: dosage-dependent; phenotype D: NEE dosage–mortality risk curve U-shaped and dangerous.
Schinkel M, 2021 [67]	Sepsis	PHANTASi	Early administration of antibiotics vs. control	Three clusters based on age, heart rate, and temperature	Significant interaction between age and benefits of early antibiotics
Neyton LPA, 2024 [50]	Septic shock	VANISH	Hydrocortisone vs. placebo	SRS1 and SRS2	SRS2 and hydrocortisone: increased mortality
Neyton LPA, 2024 [50]	Septic shock	VANISH	Hydrocortisone vs. placebo	Hyperinflammatory and hypoinflammatory	Hypoinflammatory and hydrocortisone: increased mortality
Rajendran S, 2025 [74]	Sepsis	Observational data	Steroids	Rapidly improving and rapidly worsening subgroup trajectory	Rapidly improving and steroids: increased mortality and longer time to ICU discharge and duration of ventilation
Kudo D, 2021 [75]	Sepsis	Observational data	Recombinant human thrombomodulin	Clusters dA (more pronounced coagulopathy), dB, dC, dD	Cluster dA and thrombomodulin: decreased mortality
Bruse N, 2024 [77]	Sepsis-associated AKI	REVIVAL	Ilofotase alfa vs. placebo	Phenotype 1 (less severe) and phenotype 2 (more critically ill)	Phenotype 2 and ilofotase alfa: less major adverse kidney events up to day 90
Fish M, 2022 [78]	COVID-19	REMAP-CAP	Convalescent plasma therapy vs. usual care	Subphenotype 1 (elevated type II and II effector immune responses), 2 (exaggerated inflammation), and 3 (variable biomarker patterns)	Subphenotype-1 and convalescent plasma therapy: fewer organ support-free days

Abbreviations: AKI, acute kidney injury; ARDS, acute respiratory distress syndrome; ICU, intensive care unit; NEE: norepinephrine equivalence; PEEP, positive end-expiratory pressure; SRS, sepsis response signature.

## 4. Discussion

This review highlights AI/ML-derived sepsis subphenotypes and endotypes, as promising tools for the improved understanding of heterogeneity in sepsis biology, which is essential in diagnosis, prognosis, and response to treatment. AI/ML-derived subphenotypes share several common and reproducible features, validating their existence across different cohorts, using different models and input data. Despite the multiplicity of clustering efforts, a predominantly hyper- (including features of endotheliopathy and coagulopathy) and a hypoinflammatory pattern is repeatedly recognized, as opposed to adaptive profiles with least alterations [79]. The most promising subphenotyping efforts are the ones that are validated across different cohorts and provide a strong biological base for their existence: the sepsis clinical phenotypes (α-, β-, γ-, δ-) [12], the molecular ARDS or sepsis phenotypes [44], and the temperature trajectories [25] are examples of promising subphenotypes to be used as predictors of outcome or treatable traits. Endotypes with overlapping features, such as SRS1, MARS 2, and inflammopathic [55], or bridges to subphenotypes, such as SRS2 and hyperinflammatory or SRS1 and hypoinflammatory molecular phenotype [50], are also very appealing and require further study as prospective classifiers. There is increasing research on AI use in decision-making assistance and prediction model construction in the ICU and ED [9]. In clinical practice, the sepsis Immunoscore is an FDA-approved AI/ML tool, using input from the EHR to stratify patients into different risk groups for sepsis diagnosis [80]. Interestingly, models that take into account sepsis heterogeneity based on initial phenotyping, perform better for sepsis prognosis than a general model, predicting overall deterioration [81,82].

The main implication of these subphenotypes lies in their impact on sepsis management. However, significant questions remain. Underlying biological pathways and networks, driving these hyper- and hypo-inflammatory patterns, remain poorly understood, and mortality is not consistently higher in one pattern (e.g hyperinflammatory), while other known biological mechanisms, such as resilience or resistance, are missing [83]. Clinical phenotypes and signature-based endotypes show little overlap or overtime stability [50,84,85]. A major drawback is that subphenotyping studies rely mainly on retrospective data, with limited generalizability, due to scarce weighting in different geographical locations [86]. The results need validation in large prospective datasets consisting of populations with different characteristics. Moreover, the information carried by each clustering effort should be better integrated into larger international consensus frameworks, such as Consensus Transcriptomic Subtyping (CTS) or SUBSPACE, as described above [55,61]. As an example, models built in the SENECA derivation cohort to predict membership to α-, β-, γ-, and δ- (clinical) phenotypes were tested for validation in four other cohorts, MARS, MARS2, NICE, and MIMIC-IV consisting of 52,226 sepsis patients. The phenotype distribution and in-hospital mortality rates in differed among the four cohorts for the α, γ, and δ types [87]. Thus, subphenotyping to achieve predictive enrichment in immunotherapy trials is still in its infancy. The results are prone to bias, and apart from the ongoing RECORDS RCT on corticosteroids for septic shock, discussed above, no other trial is using ML-derived clusters prospectively to apply immunotherapy. Moreover, the actual treatment response can vary considerably between individuals and can differ substantially from the average treatment effect. Statistical approaches have been developed to analyze the heterogeneity of the treatment effect and to predict individualized treatment effects for each patient [88]. Moreover, ethical considerations in conducting AI-assisted prospective trials may arise; data privacy and protection should be better addressed through encryption techniques and policy regulations; AI misuse, and inequity should be expected, especially due to the underrepresentation of certain social–ethnic or economic groups in training sets. Thus, when designing AI-assisted prospective trials, a step of comparison with traditional (clinical judgment-assisted) methods of enrollment may need to be undertaken, to avoid issues of data breach or inequity and to correctly identify groups of patients in need of a specific intervention.

AI and the use of ML techniques offer an unprecedented opportunity to integrate large complicated data into simpler, rapid, and ready-to-use classifiers. There are a number of real-world restrictions and limitations relative to AI/ML methods for the precision management of sepsis. As with conventional statistical approaches, ML clustering algorithms can be sensitive to missing data, particularly when data are not missing completely at random, and imputations are applied. Dataset shift, non-representativeness of the general population, and the population variability may be falsely and overly interpreted as specific patterns by AI. Determining the optimal number of clusters and selection of the most appropriate clustering algorithm are of paramount importance but remain non-standardized. Inherent algorithmic bias should better be addressed in prospective comparative studies to select the most appropriate model or model combination [89,90,91,92]. Moreover, clustering algorithms are highly influenced by the quality of the data fed into them, necessitating careful selection of the included variables; and in parallel, the maximum number of variables that can be used in clustering is limited by the sample size. Preselection of candidate variables may be helpful, and those that reflect underlying disease mechanisms and pathophysiological processes are preferable.

In the era of AI, implementation of subphenotyping in clinical routine requires overcoming several barriers: algorithms and clusters should be easily measurable, immune endotypes should be coupled with easy-to-use assays, and new endotypes and assays-biomarkers should be accessible in different countries, even in settings with limited resources [68]. Thus, subphenotyping may even be embedded in the definition of clinical syndromes like sepsis, as was the case for ARDS after an international Delphi expert panel consensus to refine the definitions to better account for the heterogeneity of clinical presentations and underlying pathophysiology and to improve the diagnostic precision [93].

## 5. Conclusions

The AI/ML-derived classification of sepsis, together with an improved understanding of sepsis biology, are powerful tools for understanding the heterogeneity in the treatment response. A number of promising subphenotypes and endotypes have been identified, such as the clinical sepsis phenotypes, the ARDS molecular phenotypes, and the consensus transcriptomic subtypes, that require further consensus and prospective validation in large international datasets. The specific underlying biological processes behind these subphenotypes remain unknown, limiting their use as treatable traits for immunotherapy, but this remains to be tested in prospective interventional clinical trials, such as the RECORDS trial. The rational use of AI in research and clinical practice is required to successfully link specific patients with the optimal interventions.

## Data Availability

No new data were created or analyzed in this study. Data sharing is not applicable to this article.

## Abbreviations

The following abbreviations are used in this manuscript:

AI	Artificial intelligence
AKI	Acute kidney injury
ARDS	Acute respiratory distress syndrome
ARF	Acute respiratory failure
CAP	Community-acquired pneumonia
CI	Confidence interval
CTS	Consensus transcriptomic subtype
DrotAA	Drotrecogin alfa activated
ED	Emergency Department
EHR	Electronic health record
HAP	Hospital-acquired pneumonia
HCA	Hierarchical cluster analysis
HIV	Human immunodeficiency virus
HLA	Human leucocyte antigen
ICU	Intensive care unit
IL	Interleukin
LCA	Latent class analysis
LPA	Latent profile analysis
ML	Machine learning
MOD	Multi-organ dysfunction
NEE	Norepinephrine equivalence
PCA	Principal component analysis
PDW	Platelet distribution width
PEEP	Positive end-expiratory pressure
RCT	Randomized clinical trial
rhTM	Recombinant human thrombomodulin
SOFA	Sequential organ failure assessment
SRS	Sepsis response signature
sTNFR1	Soluble tumor necrosis factor receptor one
WBC	White blood cells
